# The failure of biological treatment in axial spondyloarthritis is linked to the factors related to increased intestinal permeability and dysbiosis: prospective observational cohort study

**DOI:** 10.1007/s00296-024-05614-4

**Published:** 2024-05-14

**Authors:** Magdalena Chmielińska, Anna Felis-Giemza, Marzena Olesińska, Agnieszka Paradowska-Gorycka, Dariusz Szukiewicz

**Affiliations:** 1 Department of Biophysics, Physiology and Pathophysiology, Faculty of Health Sciences, Medical University of Warsaw, 02-004 Warsaw, Poland; 2 Department of Outpatient Clinics, National Institute of Geriatrics, Rheumatology and Rehabilitation, 02-637 Warsaw, Poland; 3 Biologic Therapy Center, National Institute of Geriatrics, Rheumatology and Rehabilitation, 02-637 Warsaw, Poland; 4 Department of Connective Tissue Diseases, National Institute of Geriatrics, Rheumatology and Rehabilitation, 02-637 Warsaw, Poland; 5 Department of Molecular Biology, National Institute of Geriatrics, Rheumatology and Rehabilitation, 02-637 Warsaw, Poland

**Keywords:** Biological therapy, Haptoglobins, Intestinal barrier function, Spondylitis, Ankylosing, Treatment failure, Zonulin

## Abstract

**Background:**

A significant number of patients with axial spondyloarthritis (axSpA) do not respond to biological therapy. Therefore, we decided to investigate the specificity of this group of patients and, in particular, whether haptoglobin (Hp), its polymorphism and zonulin, in addition to other clinical features, are predictors of poor response to biological treatment.

**Methods:**

48 patients with axSpA who were unsuccessfully treated with standard drugs were converted to biological treatment, and from this time on, a 12-week follow-up was started to assess the failure of biological treatment (Bath Ankylosing Spondylitis Disease Activity Index (BASDAI) decrease < 2 points). Predictors of treatment failure were identified using logistic regression analysis.

**Results:**

21% of subjects had biological treatment failure. Patients who had a higher zonulin level, a history of frequent infections, were older, had inflammatory bowel disease (IBD), had a lower Hp level at the time of inclusion in biological therapy showed an increased risk of treatment failure.

**Conclusions:**

The results of the study support the hypothesis that the effectiveness of biological treatment of axSpA is limited by changed microbiota and intestinal epithelial barrier dysfunction, as an increased risk of biological treatment failure was observed in patients who were older, had higher zonulin level, IBD and repeated courses of antibiotics due to frequent infections. Therefore, starting biological treatment should be followed by reducing intestinal permeability and regulating the disturbed gut microbiome.

**Supplementary Information:**

The online version contains supplementary material available at 10.1007/s00296-024-05614-4.

## Introduction

Currently, the therapeutic management for axial spondyloarthropathy follows the guidelines of Assessment of SpondyloArthritis International Society-European Alliance of Associations for Rheumatology (ASAS-EULAR), which recommends the inclusion of treatment with biological or targeted synthetic disease-modifying antirheumatic drugs (b/tsDMARDs) after treatment failure with non-steroidal anti-inflammatory drugs (NSAIDs) and a positive rheumatologist’s opinion on b/tsDMARD treatment [1].

According to available research data, approximately 40–65% of patients do not respond to biological treatment, depending on the defined outcome measures, the drug used and the treatment duration [2–5]. The remission rate is even lower, although it is rarely used as an endpoint in trials, probably because it is much more difficult to achieve [5]. In addition, the initial successful treatment often becomes ineffective after some time, forcing a change of therapy [6].

There is a number of studies on predictors of a good response to biological treatment [7–9]. The ASAS-EULAR recommendations list C-reactive protein (CRP) and MRI sacroiliitis as factors that increase the likelihood of response to tumour necrosis factor inhibitors (TNFi) [1]. Thus, the rheumatologists better identify the group of patients for whom this treatment modality has the highest chance of success. For patients without these predictive factors, the choice of biological treatment is more difficult but, in the absence of an NSAIDs effect, probably the only option, along with tsDMARDs.

In our study, we changed the previous way of approaching the problem of treatment choice after failed NSAID therapy. We focused on the potential factors related to the resistance to bDMARDs.

Based on existing data on the pathogenesis of axSpA, we decided to expand the search for predictors of response to biological treatment to include variables that were not previously considered as potential predictors: the variable associated with inflammation (Hp), related to increased intestinal permeability (zonulin, IBD) and disturbed intestinal flora (repeated courses of antibiotics due to frequent infections) [10–13]. We necessarily included the Hp polymorphism because the structure of the Hp molecule and its functional properties are polymorphism-dependent [14].

Especially since many data indicate that Hp 2–2 is a phenotype associated with a worse course of certain diseases, including autoimmune disorders [15–18].

Given that a high proportion of patients have normal CRP and erythrocyte sedimentation rate (ESR), we considered that another inflammatory marker might prove to be a better predictor of response to treatment.

Hp is not only an acute-phase protein, but is also involved in modulating the response of immune cells to various cytokine signals associated with inflammation and the lipopolysaccharide response [19]. On the other hand, zonulin itself is a precursor of Hp2—as the first eukaryotic member of the zonulin family peptides (ZFP) [20]. Existing enzyme-linked immunosorbent assays (ELISAs) for zonulin, however, also detect other proteins from ZFP [21]. Zonulin appears to be associated with the gut-joint axis and in our last report we demonstrated its association with poor response to NSAIDs [22].

The aim of this study was to investigate which of the selected clinical features of axSpA extended by Hp concentration, its polymorphism and zonulin concentration predict biological treatment failure.

## Materials and methods

### Study design

**Fig. 1:**
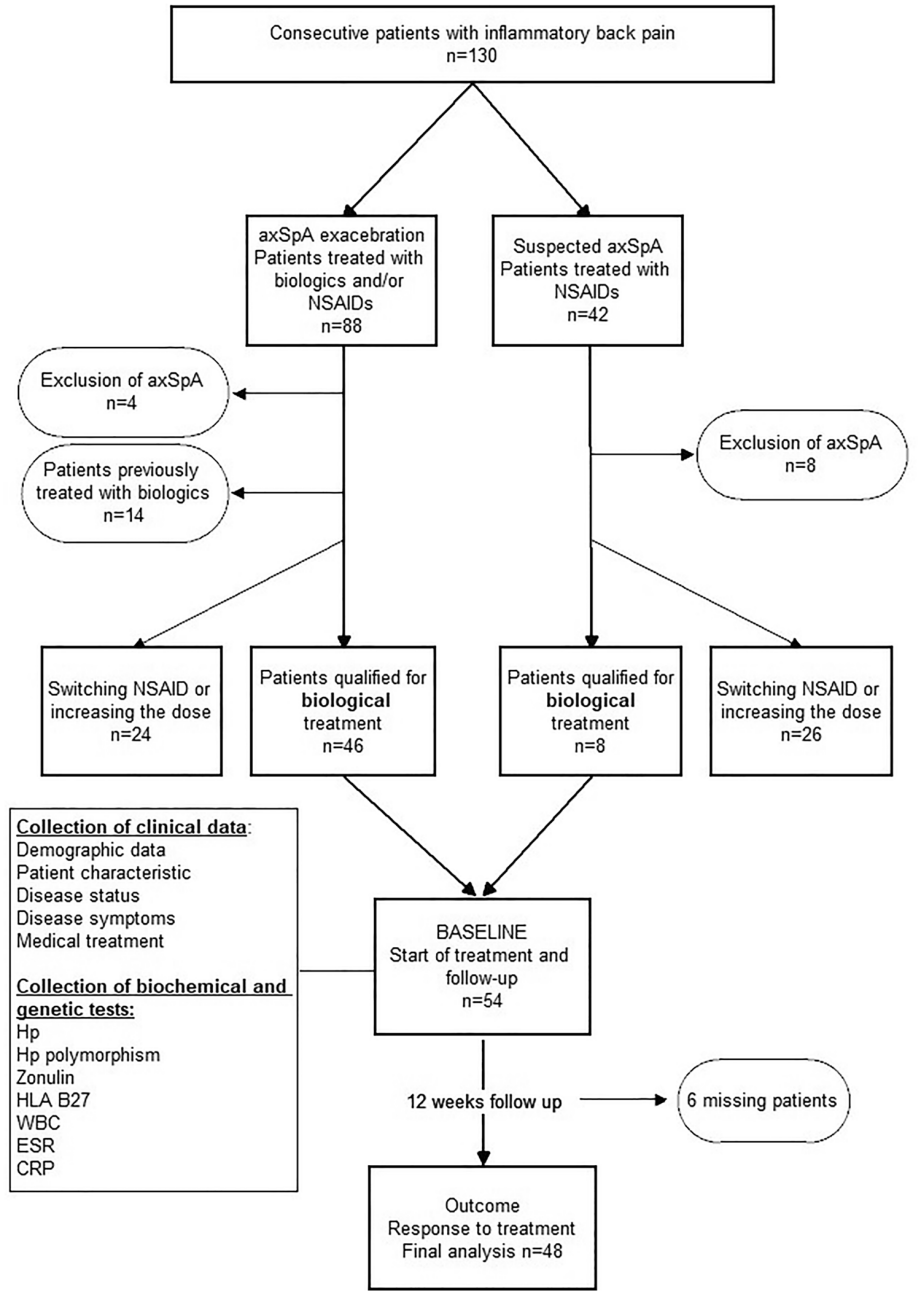
Flowcharts of study design and patients enrolment process. *axPsA* axial psoriatic arthritis, *BASDAI* Bath Ankylosing Spondylitis Disease Activity Index, *CRP-C* reactive protein, *ESR* erythrocyte sedimentation rate, *HLA-B27* human leukocyte antigen B27, *Hp* haptoglobin, *NSAIDs* non-steroidal anti-inflammatory drugs, *WBC* white blood count

The prospective observational cohort study included patients with axSpA converted to biological treatment after failure of NSAIDs therapy according to ASAS-EULAR recommendations [1]. The consecutive sampling patients hospitalized between November 2020 and October 2022 at the National Institute of Geriatrics, Rheumatology and Rehabilitation in Warsaw were recruited to the study. The study design and enrollment process is presented on the flowchart (Fig. 1). The diagram is a part of the flowchart from our previous study and reflects its section on biological treatment [22]. All participants signed an informed consent. The study received positive approval from the Bioethics Committee at the National Institute of Geriatrics, Rheumatology and Rehabilitation in Warsaw (Date: 23 October 2020; No KBT- 5/1/2020).

### Sample size

The sample size was calculated based on the following assumptions:

- 1. The ratio of patients with an elevated CRP ( +) and a normal CRP (−) who will be admitted to the hospital for spinal inflammatory back pain in the course of axSpA will be 2:1.
- 2. The treatment failure rate in the CRP ( +) and CRP (−) groups will be 0.3 and 0.7, respectively.
- Assuming an alpha error will not exceed 0.05 and a test power will be at least 80%, the calculated number of patients was 66.

### Patients

54 patients with non-radiographic axSpA (nr-axSpA), axial psoriatic arthritis (axPsA), ankylosing spondylitis (AS) who met the axSpA classification criteria according to ASAS 2010 and who were qualified for biological treatment according to the ASAS-EULAR recommendations were included in the study [1, 23]. The inclusion criterion was the presence of inflammatory spinal pain as a reason for hospitalisation [24]. Exclusion criteria included conditions affecting Hp levels: active infection, haemolytic anaemia, active malignancy, concomitant other inflammatory connective tissue disease, pregnancy [25]. Additional exclusion criteria consisted of: back pain unrelated to axSpA, qualification for therapy other than bDMARDs, previous use of bDMARDs and eligibility for biological treatment for reasons other than treatment failure with NSAIDs.

### Data collection and study measures

At baseline, we collected clinical data on demographics (sex, age), patient characteristics (body mass index, SpA subtype, disease duration, family history of SpA, smoking status, history of frequent infection), disease status (disease activity measured by BASDAI, severity of back pain according to visual analogue scale (VAS) by the patient, degree of sacroiliac joint involvement on X-ray, presence of sacroiliitis on magnetic resonance imaging (MRI), presence of syndesmophytes), symptoms (arthritis, tendinitis, present and past history of uveitis, buttock pain), medical treatment (biological and conventional DMARDs, glucocorticosteroids (GCS)) and comorbidities. The selected biochemical and genetic parameters were determinated: Hp level, Hp polymorphism, zonulin level, CRP, ESR, white blood count (WBC), human leukocyte antigen B27 (HLA-B27). The data collected is detailed in Table 1. Frequent infections were defined as recurrent and requiring antibiotics.

**Table 1:** Baseline characteristics of the patients, *n* = 48

	Data missing (*n*)	*n* (%) or median (IQR)
Patients demographics		
Female, *n* (%)	0	27 (56,3)
Age, median (IQR), years	0	36.7 (30.8–44.4)
Patient characteristic		
BMI, kg/m^2^	1	24.3 (17.3–33.5)
axPsA, *n* (%)	0	14 (29.2)
AS, *n* (%)	0	27 (56.3)
nr-axSpA, *n* (%)	0	7 (14.6)
HLA B27 positivity, *n* (%)	0	38 (79.2)
Hp 1–1 phenotype, *n* (%)	3	10 (22.2)
Hp 1–2 phenotype, *n* (%)	3	20 (44.4)
Hp 2–2 phenotype,* n* (%)	3	15 (33.3)
Symptom duration, median (IQR), years	0	8 (4–15.5)
Years since diagnosis, median (IQR), years	0	1.2 (0.2–3.5)
Family history of SpA,* n* (%)	0	8 (16.7)
Active or past smokers, *n* (%)	0	6 (12.5)
History of frequent infections, * n* (%)	0	12 (25)
Disease status		
BASDAI, median (IQR)	0	6.9 (5.6–8.0)
VAS, median (IQR), mm	1	71 (60–82)
MRI sacroiliitis, *n* (%)	30	12 (66.7)
Syndesmophytes, *n* (%)	0	7 (14.6)
x-ray sacroiliitis		
No changes, *n* (%)	0	5 (10.4)
x-ray sacroiliitis of ≥ 1 joint in grade:	0	1 (2.1)
≥ 1, *n* (%) (0–1)		
≥ 2, *n* (%) (1–2)	0	22 (45.8)
≥ 3, *n* (%) (2–3)	0	15 (31.3)
≥ 4, *n* (%) (3–4)	0	4 (8.3)
Disease symptoms		
Arthritis, *n* (%)	0	15 (31.3)
Tendinitis, *n* (%)	0	10 (20.8)
Uveitis, *n* (%)	0	2 (4.2)
Uveitis ever, *n* (%)	0	12 (25)
Buttock pain, *n* (%)	1	26 (55.3)
Laboratory analyses		
ESR, median (IQR), mm/h	0	13 (7–29)
ESR, ranges, mm/h	0	2.0–87.0
CRP, median (IQR), mg/l	0	9 (5–18)
CRP, ranges, mg/l	0	1–163
WBC, median (IQR), 10 9 /L	0	7 (5.5–8.5)
WBC, ranges, 10 9 /L	0	3.4–11.1
Haptoglobin, median (IQR), mg/dl	17	381.6 (233.7–512.2)
Haptoglobin, ranges, mg/dl	17	132.5–980.8
Zonulin, median (IQR), ng/ml	0	40.5 (25.0–55.2)
Zonulin, ranges, ng/ml	0	11.8–105.4
Biological treatment		
Anty-TNF overall, *n* (%)	0	38 (79.2)
Adalimumab, *n* (%)	0	23 (47.9)
Etanercept, *n* (%)	0	3 (6.3)
Certolizumab, *n* (%)	0	9 (18.8)
Golimumab, *n* (%)	0	3 (6.3)
Secukinumab, *n* (%)	0	8 (16.7)
Ixekizumab, *n* (%)	0	2 (4.2)
Other medication		
NSAIDs, *n* (%)	0	48 (100)
cDMARDS, n (%)	0	21 (43.8)
GCS, n (%)	0	5 (10.4)
Concomitant disease		
Overall, *n* (%)	0	40 (83.3)
IBD, *n* (%)	0	10 (20.8)
Gastrointestinal symptoms, *n* (%)	0	18 (37.5)

*Anti-TNF therapy* anti tumor necrosing factor therapy (adalimumab, certolizumab, etanercept, golimumab), *AS* ankylosing spondylitis, *axPsA* axial psoriatic arthritis, *BASDAI* Bath Ankylosing Spondylitis Disease Activity Index, *BMI* body mass index, *bDMARDs* biological disease-modifying antirheumatic drugs, *cDMARDs* classic disease-modifying antirheumatic drugs, *CRP C*-reactive protein, *ESR* erythrocyte sedimentation rate, *GCS* glucocorticosteroids, *Hp* haptoglobin, *IBD* inflammatory bowel disease, *IQR* inter-quartile range, *nr-axSpA* non-radiographic axial spondyloarthritis, *MRI* magnetic resonance imaging, *NSAIDs* non-steroidal anti-inflammatory drugs, *SpA* spondyloarthritis general, *WBC* white blood count, *VAS* value of spinal pain intensity on visual analogue scale

Disease activity was assessed with BASDAI which is an instrument routinely used in clinical practice based on patient self-scoring on the severity of various ailments such as spinal and peripheral joint pain, discomfort in pressure-sensitive areas, morning stiffness and fatigue. Each question is scored on a scale of 0–10, where 10 represents the most severe complaints. BASDAI is calculated using a formula and scores >  = 4 are considered high disease activity [26]. Some patients had a sacroiliac joint MRI ordered if there was doubt about the nature of their back pain. The majority of patients, however, had this examination already performed previously but at different times and sometimes outside of the research site, therefore the results of these examinations were not included in our analysis. MRI sacroiliitis was graded as positive or negative according to the ASAS classification [27]. All patients had their sacroiliac joint structural damage assessed by X-ray. Sacroiliitis x-ray grading was according to the New York criteria [28]. Additional differential diagnosis of spinal pain was also performed.

### Biological treatment

Dosing of individual bDMARDs was according to the summary of product characteristics: adalimumab 40 mg every 2 weeks; etanercept 50 mg every week; golimumab 50 mg every month; certolizumab: first 3 doses 400 mg every 2 weeks, then 200 mg every 2 weeks; ixekizumab: first dose 160 mg, then 80 mg every 4 weeks; secukinumab: first 5 doses 150 mg per week, then 150 mg per month.

In our study, IBD should be regarded as a concomitant disease not a reason for implementing biological treatment. For IBD requiring biological treatment, patients are managed by gastroenterology departments. None of our patients required biological treatment for IBD. No patient required an increase in the dose of secukinumab due to severe psoriasis.

### Outcome

After 12 weeks of biological treatment, each patient had the disease activity assessed using BASDAI scale by completing a questionnaire on a website specially prepared for this study.

Biological treatment failure was defined as a decrease in BASDAI of less than 2 points according to the ASAS-EULAR criteria [1].

### Serum analysis for haptoglobin, haptoglobin polymorphism and zonulin

#### Haptoglobin

Serum Hp concentrations (ng/ml) in patients were measured by ELISA (Aviva Systems Biology, San Diego, CA, USA)) according to the manufacturer's instructions. The detection limit of human Hp was 0.8196 ng/ml. Each sample was tested twice, and the intra-assay coefficient of variation was 4.571%. The plates were read at 450 nm absorbance on an LT-4000MS reader (Labtech International Ltd, UK). The concentration was determined after fitting a linear standard curve as recommended in the manual.

#### Haptoglobin polymorphism

Genomic DNA was extracted from 200 µL of whole blood samples from 48 patients while using a Blood DNA Mini kit (A&A Biotechnology, Poland) following the manufacturer’s instructions. Hp-1–Hp2 polymorphism was detected by the allele‐specific PCR. For the Hp-1- and Hp2-specific sequences amplification, primer A sequence was 5′-GAGGGGAGCTTGCCTTTCCATTG-3′ and primer B sequence was 5′-GAGATTTTTGAGCCCTGGCTGGT-3′′. For the Hp2-specific sequence amplify, we used the primers: C 5′-CCTGCCTCGTATTAACTGCACCAT-3′ and D 5′-CCGAGTGCTCCACATAGCCATGT-3′. Reaction mixture contained: 50 ng of genomic DNA, 10 pmol of each primers, and Taq PCR Master Mix (EURx, Gdanska, Poland). Reaction condition for primer AB was as follows: 95 °C for 1 min, 35 cycles of 95 °C for 1 min, 66 °C for 1 min, 72 °C for 3 min and a final extension at 72 °C for 7 min. PCR products were separated on a 1.8% agarose gel containing ethidium bromide, and the Hp genotypes were determined by observing the DNA fragments under UV light.

#### Zonulin

Enzyme-linked immunosorbent assay kits (ELISA) for zonulin was performed with commercially available ELISA kits (Immunodiagnostik AG, Bensheim, Germany) according to the manufacturer's instructions. Samples from patients were separated from peripheral venous blood at room temperature and stored at − 86 °C until analysis. The minimum detection level of 0.183 ng/ml was used. Serum zonulin concentrations were detected at a wavelength of 450 nm using the microplate reader (El × 800, BIO-TEK Instruments).

#### Statistics

Descriptive data are presented using means of medians (IQR) and percentages when referring to quantitative and qualitative variables, respectively. The Spearman correlation analysis was used to assess relationships between quantitative variables, Fisher’s exact test to analyse associations between qualitative variables and the Mann–Whitney test to find relations between qualitative and quantitative variables. To assess significance the P values were used, and to estimate the strength of the associations the Spearman correlation coefficient was applied. A p-value of less than 0.05 was considered significant for all tests. To identify significant factors contributing to poor response to bDMARDs, univariate logistic regression was performed. In addition, the series of two-factor analyses with baseline variables were conducted to investigate the zonulin factor as an independent predictor of treatment failure. To estimate effect sizes odds ratios (OR) and the probability of treatment failure were used. For quantitative variables, the risk of treatment failure is presented as a curve in the graph, where the vertical axis shows the probability of a poor response. For qualitative variables, the risk of treatment failure is presented in the table, where the minus sign refers to the reference group for the given factor (control group, OR = 1.00). Odds ratios for quantitative variables are calculated per unit for each variable. Statistical analysis and data collection were performed with the SAS System (SAS/STAT® User’s Guide. Cary, NC. 2023).

## Results

Of 48 participants sufficient data were available. Patient characteristics are listed in Table 1. The majority of patients were AS patients (56.3%) and the most used bDMARDs were iTNF (79.2%) and adalimumab (47.9%). At baseline all patients were taking NSAIDs and 43.8% (*n* = 21) of them used additionally conventional disease-modifying drugs (cDMARDs) because of concomitant peripheral arthritis, tendinitis or uveitis. The doses of taken cDMARDs were as follows: methotrexate 10 mg–25 mg per week, sulfasalazine 2 g–3 g per day, one person was taking leflunomide 20 mg per day. 10.4% (*n* = 5) of subjects were using GCS at baseline due to active arthritis at doses of 4–16 mg per day prescribed to be gradually reduced and discontinued within a few weeks. The doses of cDMARDs did not differ between responders and non-responders. The use of cDMARDs or GCS at baseline increased the risk of poor response to biological treatment, but the result was not statistically significant (OR = 3.58, 95% CI 0.80 − 16.05, *p* = 0.096).

Few patients had an MRI of the sacroiliac joints (37.5%) and, of these, a positive was described in 66.7%. The majority of patients had an elevated CRP (71%). None of our patients had exacerbated psoriatic lesions or symptoms of IBD exacerbation.

The median Hp concentration was 381.6 (233.7–512.2) mg/dl and zonulin was 40.5 (25.0–55.2) ng/ml. The distribution of the Hp polymorphism was as follows: Hp1-1: 22%, Hp2-1: 44.4%, 2–2: 33.3%. Hp concentrations varied according to Hp phenotype and were significantly highest in subjects with the Hp1-1 phenotype (median Hp level,mg/dl: Hp1-1: 497.9 (387.8–650.9); Hp2-1: 436.7 (355.4–526.0); Hp2-2: 190.8 (162.1–305.1); *p* = 0.0025). Hp polymorphism was not associated with parameters of inflammatory activity, disease activity and zonulin level (Table 1S).

As in the previous study [21], we recorded the highest zonulin levels in individuals with the Hp1-1 phenotype, but the differences were not statistically significant *p* = 0.93 (median zonulin concentrations, ng/ml: Hp1-1: 42.6 (25.3–56.4); Hp2-1: 41.8 (28.7–55.8); Hp2-2: 41.2 (26.5–55.2)). The presence of zonulin in those with the Hp1-1 phenotype demonstrates that commercially available ELISAs detect other ZFP family proteins than just pre-Hp2. Zonulin was not correlated with Hp (*r* = -0.004, *p* = 0.98) and ESR (*r* = 0.11, *p* = 0.46) but it was significantly correlated with CRP (*r* = 0.3; *p* = 0.045). Zonulin levels were not significantly different in patients with IBD or current gastrointestinal symptoms.

**Fig. 2:**
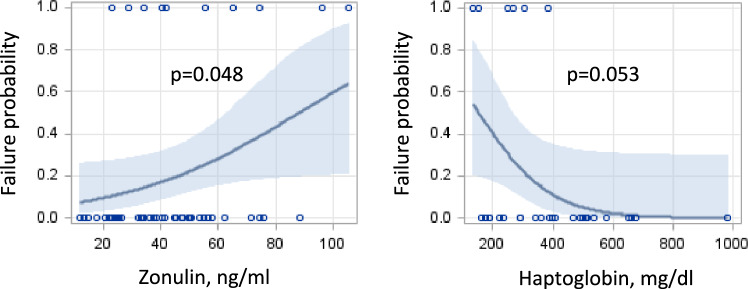
Prediction of biological treatment failure by zonulin and haptoglobin concentrations at admission to the hospital

Approximately 21% of subjects had treatment failure (baseline BASDAI decline < 2 scores). Predictors increasing the risk of biological treatment failure were previous history of frequent infections (OR = 4.43, 95% CI 1.00–19.58, *p* = 0.049) and higher zonulin levels (per 10 ng/ml OR = 1.39, 95% CI 1.02–2.00, *p* = 0.048), which remained significant after adjusting for the majority of potential confounders (Table 2) and (Table 3). Figure 2 shows the response to biological treatment depending on zonulin and Hp levels. Good response to bDMARDs was greater in those who had higher levels of Hp (per 200 mg/dl OR = 0.19, 95% CI 0.02–0.76, *p* = 0.053). All subjects who had treatment failure to bDMARDs had Hp levels below 400 mg/dl. Hp was not associated with either ESR (*r* = 0.17, *p* = 0.36) or CRP (*r* = 0.27, *p* = 0.14).

**Table 2:** Baseline predictors of treatment failure to bDMARDs

Variable	Univariate analyses, OR (95% CI)
Gender (female vs male)	0.73 (0.18 − 2.94)
Age (per 10 years)	1.88 (0.90 − 3.96)
BMI	1.14 (0.96 − 1.35)
Symptom duration (per 5 years)	1.44 (0.94–2.22)
Family history of SpA (ref negative)	1.33 (0.23 − 7.89)
History of frequent infections (ref negative)	4.43 (1 − 19.58)
Concomitant diseases (ref negative)	2.03 (0.22 − 18.77)
Treatment with cDMARDs or GCS (ref negative)	3.58 (0.80 − 16.05)
Biological treatment (anti-TNF therapy vs other bDMARDs)	0.54 (0.11 − 2.65)
Gastrointestinal symptoms (ref negative)	3.25 (0.77 − 13.69)
AS	0.73 (0.18 − 2.94)
nr-axSpA	*
axPsA	3.22 (0.76 − 13.71)
History of uveitis (ref negative)	0.7 (0.13 − 3.87)
Buttock pain (ref negative)	1.28 (0.31 − 5.28)
IBD (ref negative)	3.56 (0.77 − 16.53)
x-ray sacroiliitis	*
x-ray sacroiliitis of ≥ 1 SI joint in grade ≥ 3	1.02 (0.25 − 4.24)
Uveitis (ref negative)	4.11 (0.23 − 72.21)
Arthritis (ref negative)	0.93 (0.20 − 4.23)
Tenditis (ref negative)	0.94 (0.17 − 5.31)
HLAB27 (ref negative)	0.28 (0.06 − 1.31)
MRI sacroiliitis	*
WBC (10 9 /L)	0.66 (0.41–1.04)
ESR (mm/h)	0.95 (0.89 − 1.02)
CRP (mg/l)	0.98 (0.93 − 1.04)
CRP > 5 (mg/l)	0.54 (0.13 − 2.30)
BASDAI (1 score)	0.82 (0.50 − 1.32)
VAS (mm)	0.99 (0.95 − 1.04)
Haptoglobin (mg/dl)	0.99 (0.98 − 0.99)
Haptoglobin (per 200 mg/dl)	0.19 (0.02 –0.76)
Zonulin (ng/ml)	1.03 (1.00 − 1.07)
Zonulin (per 10 ng/ml)	1.39 (1.02 − 2.0)
Haptoglobin phenotype (Hp 2–1 vs Hp 1–1)	0.41 (0.07–2.56)
Haptoglobin phenotype (Hp 2–2 vs Hp 1–1)	0.85 (0.14 − 5.0)

*Anti-TNF therapy* anti tumor necrosing factor therapy (adalimumab, certolizumab, etanercept, golimumab), *AS* ankylosing spondylitis, *axPsA* axial psoriatic arthritis, *BASDAI* Bath Ankylosing Spondylitis Disease Activity Index, *BMI* body mass index, *bDMARDs* biological disease-modifying antirheumatic drugs, *cDMARDs *classic disease-modifying antirheumatic drugs, *CRP C*-reactive protein, *CRP* > *5 mg/l* is deemed to be increased, *ESR* erythrocyte sedimentation rate, *GCS* glucocorticosteroids, *Hp*-haptoglobin, *IBD* inflammatory bowel disease, *nr-axSpA* non-radiographic axial spondyloarthritis, *MRI* magnetic resonance imaging, *NSAIDs* non-steroidal anti-inflammatory drugs, *SpA* spondyloarthritis general, *WBC* white blood count, *VAS* value of spinal pain intensity on visual analogue scale

^*^Variables where logistic regression calculations could not be performed. See also Table 2S

**Table 3:** Predictors of treatment failure to bDMARDs in series of two-factor analyses with zonulin and other baseline variables

Zonulin OR (95% CI)		Variable OR (95% CI)		
1.04	(1.00 − 1.07)	Female	1.18	(0.25 − 5.70)
1.04	(1.01 − 1.08)	Age	1.09	(1.00 − 1.18)
1.04	(1.00 − 1.07)	BMI	1.14	(1.00 − 1.21)
1.04	(1.01 − 1.08)	Symptom duration	1.10	(1.00 − 1.21)
1.03	(1.00 − 1.07)	Family History of SpA	0.98	(0.14 − 6.83)
1.05	(1.01 − 1.09)	History of frequent infections	8.63	(1.43 − 52.21)
1.04	(1.00 − 1.07)	Concomitant diseases	2.29	(0.21 − 25.09)
1.04	(1.00 − 1.07)	Treatment with DMARDs or GCS	3.77	(0.77 − 18.41)
1.04	(1.00 − 1.07)	Treatment with anti-TNF	0.50	(0.09 − 2.74)
1.03	(1.00 − 1.07)	Treatment with other bDMARDs	1.70	(0.38 − 7.62)
1.04	(1.00 − 1.08)	Gastrointestinal symptoms	4.63	(0.91 − 23.48)
1.04	(1.00 − 1.07)	AS	0.49	(0.10 − 2.32)
1.03	(1.00 − 1.07)	nr-axSpA	*	*
1.05	(1.00 − 1.10)	axPsA	5.91	(0.99 − 35.1)
1.04	(1.00 − 1.07)	History of uveitis	0.50	(0.08 − 3.22)
1.04	(1.00 − 1.08)	Buttock pain	2.09	(0.40 − 10.77)
1.05	(1.01 − 1.09)	IBD	7.56	(1.06 − 54.06)
1.03	(1.00 − 1.06)	No x-ray sacroiliitis	*	*
1.04	(1.00 − 1.07)	x-ray sacroiliitis of ≥ 1 SI joint in grade ≥ 3	0.75	(0.16 − 3.53)
1.04	(1.00 − 1.07)	Uveitis	6.99	(0.30 − 161.5)
1.04	(1.00 − 1.07)	Arthritis	0.57	(0.10 − 3.25)
1.03	(1.00 − 1.07)	Tenditis	0.87	(0.15 − 5.14)
1.04	(1.00 − 1.07)	HLA B27 ( +)	0.27	(0.05 − 1.37)
1.07	(0.95 − 1.20)	MRI sacroiliitis ( +)	*	*
1.03	(1.00 − 1.07)	WBC (10 9 /L)	0.69	(0.44 − 1.10)
1.04	(1.00 − 1.07)	ESR (mm/h)	0.95	(0.88 − 1.02)
1.04	(1.00 − 1.07)	CRP (mg/l)	0.96	(0.90 − 1.04)
1.04	(1.00 − 1.07)	BASDAI (1 score)	0.78	(0.47 − 1.32)
1.03	(1.00 − 1.07)	VAS (mm)	0.99	(0.95 − 1.04)
1.04	(0.98 − 1.10)	Haptoglobin (mg/dl)	0.99	(0.98 − 1.00)
1.03	(1.00 − 1.07)	Haptoglobin phenotype:		
		Hp 1–1	1.00	
		Hp 2–1	0.45	(0.07 − 3.00)
		Hp 2–2	0.90	(0.14 − 5.86)

*Anti-TNF therapy* anti tumor necrosing factor therapy (adalimumab, certolizumab, etanercept, golimumab), *AS*-ankylosing spondylitis, *axPsA* axial psoriatic arthritis, *BMI* body mass index, *BASDAI*-Bath Ankylosing Spondylitis Disease Activity Index, *bDMARDs* biological disease-modifying antirheumatic drugs, *cDMARDs*-classic disease-modifying antirheumatic drugs, *CRP-C*-reactive protein, *GCS* glucocorticosteroids, *Hp*-haptoglobin, *IBD* inflammatory bowel disease, *nr-axSpA* non-radiographic axial spondyloarthritis, *MRI* sacroiliitis-sacroiliitis visible on magnetic resonance imaging, *NSAIDs* non-steroidal anti-inflammatory drugs, other *bDMARDs* other biological disease-modifying antirheumatic drugs (iksekizumab, secukinumab), *other SpA* spondyloarthritis general, *WBC*-white blood count, *VAS*-value of spinal pain intensity on visual analogue scale

^*^Variables where logistic regression calculations could not be performed. See also Table 2S

Furthermore, in two-factor analyses with zonulin, older age (OR = 1.09, 95% CI 1.00 − 1.18, *p* = 0.047) and IBD (OR = 7.56, 95% CI 1.06 − 54.06, *p* = 0.044) have also proved to be predictors of poor response to biological treatment independently of zonulin (Table 3).

We did not report any disease activity markers (WBC, ESR, CRP, arthritis, tenditis, BASDAI, VAS) to be significantly associated with our endpoint. Although it all reduced the risk of poor response. Furthermore, all patients with active MRI sacroiliitis responded well to bDMARDs and therefore logistic regression calculations with this factor could not be performed.

Data on the incidence of treatment failure according to the different factors are included in supplement Table 2S.

## Discussion

Failure to biological treatment is an important issue and challenge for today's rheumatology. The current literature focuses on identifying predictors of good response to bDMARDs, while there is a great need to find the causes of the resistance. In addition, only known and routinely assessed markers of disease activity are usually considered for research [29–31].

In our study, we went deeper into the pathogenesis of axSpA and decided to include factors that have not been considered so far in the context of biological treatment failure. The idea to explore factors related to the microbiome and increased intestinal permeability came from the constantly arising number of reports on the importance of the gut-joints axis [32–35]. Disruption of the intestinal barrier function has been shown to predict onset of arthritis and zonulin was the main agent associated with this process [34, 36].

In addition, zonulin was shown to be up-regulated in ileal samples of patients with AS. The authors demonstrated that zonulin is able to stimulate the expansion of macrophages with the M2 phenotype, which are involved in SpA gut inflammation and synovitis [13].

Interestingly, as in our previous study, higher levels of zonulin were associated with poor response to treatment in axSpA [22]. It appears that persistently increased intestinal permeability 'interferes' with therapy. This may be due to the continuous stimulation of the immune system by intestinal antigens, probably related to dysbiosis. It is possible that the condition of intestinal damage and disturbance of the microbiome itself is linked to NSAID use [37].

This is a very exciting result, especially in the context of the study showing that the use of the zonulin antagonist larazotide acetate can inhibit arthritis [36].

The lack of association of zonulin with gastrointestinal symptoms and IBD shows difficulty in selecting individuals with increased intestinal permeability based on the medical history and clinical symptoms. Similar results have already been reported in other studies [38, 39]. It is likely that the relationship of IBD and gastrointestinal with biological treatment failure did not depend only on a damaged intestinal barrier. In our study, these two factors were zonulin-independent predictors (OR = 7.56, 95% CI 1.06 − 54.06, *p* = 0.044; OR = 4.63, 95% CI 0.91 − 23.48 *p* = 0.064, respectively). It seems that healing the gastrointestinal tract, whatever the cause, may increase the possibility of therapeutic success in axSpA.

Our hypothesis related to the influence of the microbiome on response to treatment seems to be supported by two other factors: age and the history of frequent infections, which independently of zonulin were negative predictors (OR = 1.09, *p* = 0.047; 95% CI 1.00 − 1.18, OR = 8.63, 95% CI 1.43 − 52.21, *p* = 0.019, respectively). According to the results of another study, the composition of the gut microbiota at baseline may have a better predictive value for response to TNFi than indicators of disease activity including CRP.

A history of frequent infections increased the risk of treatment failure of bDMARDs by more than eightfold! This probably may stem from the disruption of the gut microflora caused by frequent antibiotic use.

Age, on the other hand, is linked to dysbiosis [40]. Previous reports have already shown a decrease in treatment effectiveness with age in axSpA, but this was usually associated with the presence of advanced degenerative changes in the spine or a higher degree of x-ray sacroiliitis [41–43]. In our study, x-ray sacroiliitis was not associated with response to treatment, whereas age was. Age was also not correlated with the degree of x-ray sacroiliitis.

Vallier et al. demonstrated that the composition of the gut microbiota at baseline in axSpA patients presented better predictive value for response to TNFi than indicators of disease activity including CRP [44].

Also, psoriasis treatment studies have noted differences in response to bDMARDs depending on the composition of the microbiome [45, 46].

Similarly, in another study, concomitant diseases were associated with worse treatment effects with TNFi in axSpA [47]. In our study concomitant diseases did not significantly increase this risk (OR = 2.03, 95% CI 0.22 − 18.77, *p* = 0.53).

As in our previous report, zonulin was detected in all patients, not only in Hp2 antigen carriers, and, as before, was highest in those with the Hp 1–1 phenotype (*p* = 0.91), demonstrating that the ELISA detected more than just the pre-Hp2 molecule [21, 22].

Zonulin was significantly correlated with CRP, although CRP alone was not associated with treatment response. This is a different result from most studies, which have shown the superiority of increased CRP in predicting good response to bDMARDs, especially TNFi [29, 30, 47, 48]. However, higher values of the inflammatory indices (WBC, CRP, ESR) reduced the risk of poor response, but not significantly. A substantially better predictive value demonstrated Hp level.

High levels of Hp were present in patients responding well to biological therapy. In fact, Hp concentrations above 400 mg/dl were the cut-off point for responders. The result was on the borderline of significance (*p* = 0.056), which may result from the small size of the group. It is possible that, like CRP, Hp level reflects inflammation and define those who may benefit from biological treatment.

Although there were significant differences in Hp levels between phenotypes none of the phenotypes proved to be a predictor of treatment failure to bDMARDs.

MRI sacroiliitis only occurred in patients who responded well to treatment, which is in line with other study results indicating MRI sacroiliitis as a predictor of good response to standard and biological therapy [22, 30, 49, 50]. In our analysis we had a lot of missing data, the result was not statistically significant. Also, all patients without radiographic changes in the sacroiliac joints responded well, reflecting the good efficacy of biological treatment at an early stage of the disease.

A strong part of this study is its observational character, which reflects ‘real-life’ clinical situations with consecutive sampling patients.

In addition, we analysed risk factors for a poor response to bDMARDs that had never been considered until now. We shed new light on certain aspects in the approach to treating patients with axSpA and identified factors that can be modified and increase the chance of therapeutic success.

Our study had also some limitations. Due to the COVID-19 pandemic, we were unable to collect more patients and the final sample size is smaller than intended. For this reason, we did not perform separate predictors analyses for different groups of bDMARDs. Although we were not able to do multivariate analysis due to small sample size, we performed a univariate analysis with zonulin referring to the factor of most interest.

In the assessment of axSpA disease activity, we did not use the Ankylosing Spondylitis Disease Activity Score (ASDAS), which is more appropriate for this purpose. Determination of inflammatory indices after 12 weeks of biological treatment was difficult due to pandemic.

It may seem that the inclusion of only hospitalised patients in the study raises the risk of a sample selection bias. However, in Poland, diagnosis of spondyloarthritis is most often made in the hospital setting due to limitations in the operation of outpatient clinics, whereas qualification for biological treatment can only take place in hospital. Instead, hospitalisation made it possible to quickly rule out possible other causes of the back pain.

## Conclusions

In our study, we identified factors associated with intestinal dysfunction (zonulin, older age, IBD, frequent use of antibiotics) that are valuable for the prediction biological treatment failure in axSpA. We presume that by modifying the gut microbiota and/or using a zonulin inhibitor, treatment outcomes may be improved.

This is a new approach to the therapy of this disease, as it focuses on the second element of the gut-joint axis, the regulation of normal intestinal function. Whether this will help increase the effectiveness of therapy and achieve remission in SpA may only present future studies aimed at restoring homeostasis in the gut.

Furthermore, Hp appears to be, regardless of its polymorphism, a potential predictive marker of response to bDMARDs, which needs to be confirmed in further research. According to our analysis, it may prove to be a better predictor than other indices of inflammation.

### Supplementary Information

Below is the link to the electronic supplementary material.

Supplementary file1 (DOC 363 KB)

## Data Availability

The data analysed during this study are available from the corresponding author on reasonable request.
