# Supplementary material for: The failure of biological treatment in axial spondyloarthritis is linked to the factors related to increased intestinal permeability and dysbiosis: prospective observational cohort study
Source: Rheumatol Int. 2024 May 14;44(8):1487–99. doi: 10.1007/s00296-024-05614-4 (PMC11222282; doi:10.1007/s00296-024-05614-4)
Supplement: Supplementary file 1 — Supplementary file1 (DOC 363 KB) [file 296_2024_5614_MOESM1_ESM.doc]

Journal: Rheumatology International

Title: The failure of biological treatment in axial spondyloarthritis is linked to the factors related to increased intestinal permeability and dysbiosis: prospective observational cohort study

Magdalena Chmielińska^1,2*^, Anna Felis-Giemza^3^, Marzena Olesińska^4^, Agnieszka Paradowska-Gorycka^5^, Dariusz Szukiewicz^1^

^1^-Department of Biophysics, Physiology & Pathophysiology, Faculty of Health Sciences, Medical University of Warsaw, 02-004 Warsaw, Poland

^2^- Department of Outpatient Clinics, National Institute of Geriatrics, Rheumatology and Rehabilitation, 02-637 Warsaw, Poland.

^3^- Biologic Therapy Center, National Institute of Geriatrics, Rheumatology and Rehabilitation, 02-637 Warsaw, Poland.

^4^- Department of Connective Tissue Diseases, National Institute of Geriatrics, Rheumatology and Rehabilitation, 02-637 Warsaw, Poland.

^5^ -Department of Molecular Biology, National Institute of Geriatrics, Rheumatology and Rehabilitation, 02-637 Warsaw, Poland.

*Correspondence to [m.chmielinskaa@gmail.com](mailto:m.chmielinskaa@gmail.com),

Table 1S. Associations of Hp polymorphism with inflammatory markers, indicators of disease activity and zonulin

|  | **Hp 1-1** | **Hp 2-1** | **Hp 2-2** | **p** |
| --- | --- | --- | --- | --- |
| **WBC, median (IQR), 10 ^9^ /L** | 5.7 (4.9-8.3) | 7.0 (5.7-8.5) | 7.4 (6.3-9.2) | 0.39 |
| **ESR, median (IQR), mm/h** | 9.5 (6.0-19.0) | 12.0 (5.00-32.0) | 16.0 (9.0-38.0) | 0.40 |
| **CRP, median (IQR), mg/l** | 9.0 (5.0-18.0) | 6.0 (4.0-17.0) | 13.0 (5.0-26.0) | 0.37 |
| **Haptoglobin, median (IQR), mg/dl** | 498.0 (387.7-650.9) | 436.7 (335.4-526.0) | 190.8 (162.1-305.1) | 0.003 |
| **Zonulin, median (IQR), ng/ml** | 42.6 (25.3-56.4) | 41.8 (28.7-55.8) | 41.2 (26.5-55.2) | 0.93 |
| **BASDAI, median (IQR), scores** | 6.7 (5.6-7.1) | 7.4 (6.1-8.2) | 6.5 (5.6-8.0) | 0.49 |
| **VAS, median (IQR), mm** | 60.0 (57.0-80.0) | 76.5 (63.0-83.0) | 70.0 (52.0-82.0) | 0.34 |
| **BASDAI**-Bath Ankylosing Spondylitis Disease Activity Index, **CRP**- C-reactive protein, **ESR**- erythrocyte sedimentation rate, **Hp**- haptoglobin, **IQR**- inter-quartile range, **WBC**- white blood count, **VAS**- value of spinal pain intensity on visual analogue scale | | | | |

Table 2S. Incidence of failure depending on values or categories of the studied factor

| **Factors** | | |  | **% of failure** |
| --- | --- | --- | --- | --- |
| Gender | | Female |  | 18.5 |
|  |  | Male |  | 23.8 |
| Family history of SpA | | | + | 25.0 |
|  |  |  | − | 20.0 |
| History of frequent infections | | | + | 41.7 |
|  |  |  | − | 13.9 |
| Concomitant diseases | | | + | 22.5 |
|  |  |  | − | 12.5 |
| Treatment with cDMARDs or GCS | | | + | 31.8 |
|  |  |  | − | 11.5 |
| Biological  Therapy | Anti-TNF therapy | |  | 18.9 |
|  | Other bDMARDs | |  | 30.0 |
| Gastrointestinal symptoms | | | + | 33.3 |
|  |  |  | − | 13.3 |
| AS | | | + | 18.5 |
|  |  |  | − | 23.8 |
| nr-axSpA | | | + | 0 |
|  |  |  | − | 24.4 |
| axPsA | | | + | 35.7 |
|  |  |  | − | 14.7 |
| History of Uveitis | | | + | 16.7 |
|  |  |  | − | 22.2 |
| Buttock pain | | | + | 23.1 |
|  |  |  | − | 19.1 |
| IBD | | | + | 40.0 |
|  |  |  | − | 15.8 |
| x-ray sacroiliitis | | | + | 23.3 |
|  |  |  | − | 0 |
| x-ray sacroiliitis of ≥1 SI joint in grade ≥ 3 | | | + | 21.1 |
|  |  |  | − | 20.7 |
| Uveitis | | | + | 50.0 |
|  |  |  | − | 19.6 |
| Arthritis | | | + | 20.0 |
|  |  |  | − | 21.2 |
| Tendinitis | | | + | 20.0 |
|  |  |  | − | 21.1 |
| HLAB27 | | | + | 15.8 |
|  |  |  | − | 40.0 |
| MRI sacroiliitis | | | + | 25.0 |
|  |  |  | − | 0 |
| CRP> 5mg/l | | | + | 17.7 |
|  |  |  | − | 28.6 |
| Hp polymorphism | | Hp 1-1 |  | 30.0 |
|  |  | Hp 2-1 |  | 15.0 |
|  |  | Hp 2-2 |  | 26.7 |
| Age (years) | | | | 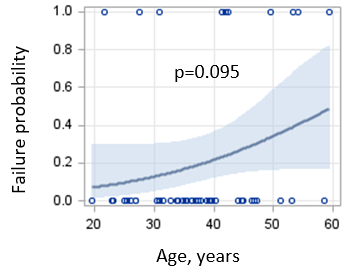 |
| Symptom duration (years) | | | | 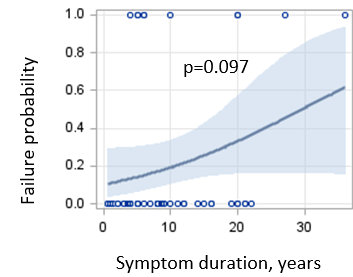 |
| WBC (10 ^9^ /L) | | | | 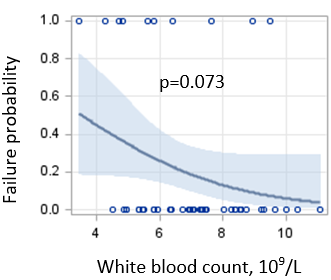 |
| ESR (mm/h) | | | | 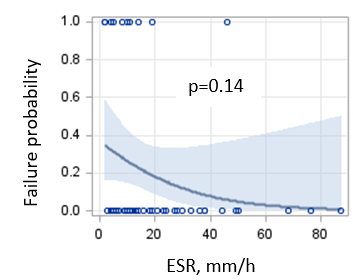 |
| CRP (mg/l) | | | | 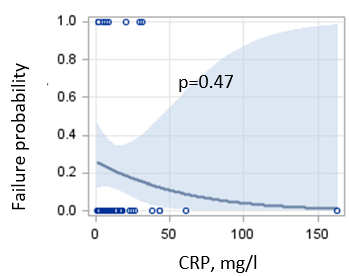 |
| BASDAI (scores) | | | | 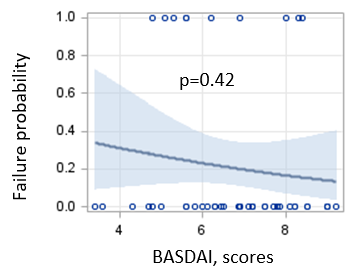 |
| VAS (mm) | | | | 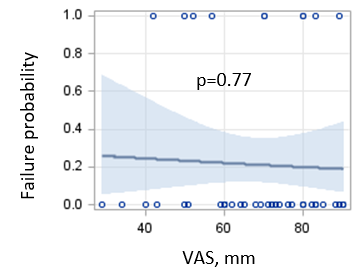 |
| Haptoglobin (mg/dl) | | | | 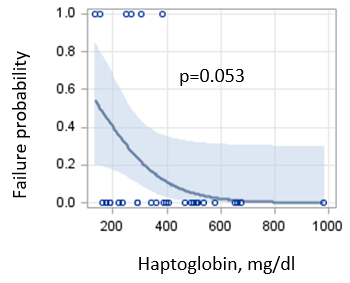 |
| Zonulin (ng/ml) | | | | 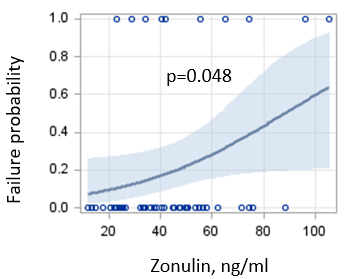 |
| BMI (kg/m^2^) | | | | 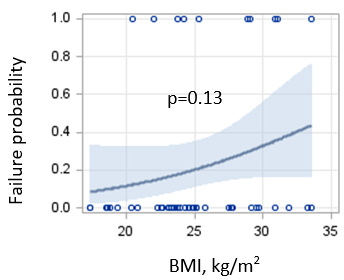 |
| **Anti-TNF therapy**- anti-tumor necrosing factor therapy (adalimumab, certolizumab, etanercept, golimumab), **AS**- ankylosing spondylitis , **axPsA**- axial psoriatic arthritis, **BASDAI**-Bath Ankylosing Spondylitis Disease Activity Index, **BMI**- body mass index, **bDMARDs**- biological disease-modifying antirheumatic drugs, **cDMARDs**- classic disease-modifying antirheumatic drugs, **CRP**- C-reactive protein, **CRP>5mg/l** is deemed to be increased, **ESR**- erythrocyte sedimentation rate, **GCS**- glucocorticosteroids, **Hp**- haptoglobin, **IBD**- inflammatory bowel disease, **IQR**- inter-quartile range, **nr-axSpA**- non-radiographic axial spondyloarthritis, **MRI**- magnetic resonance imaging, **NSAIDs**- non-steroidal anti-inflammatory drugs, **other bDMARDs**- other biological disease-modifying antirheumatic drugs (iksekizumab, secukinumab ), **SpA-** spondyloarthritis general , **WBC**- white blood count, **VAS**- value of spinal pain intensity on visual analogue scale | | | | |
